# The neurobiology of taboo language processing: fMRI evidence during spoken word production

**DOI:** 10.1093/scan/nsz009

**Published:** 2019-02-01

**Authors:** Samuel J Hansen, Katie L McMahon, Greig I de Zubicaray

**Affiliations:** 1 School of Psychology, The University of Queensland, Brisbane, QLD, Australia; 2 Herston Imaging Research Facility and School of Clinical Sciences, Queensland University of Technology, Brisbane, QLD, Australia; 3 Faculty of Health, Institute of Health and Biomedical Innovation, Queensland University of Technology, Brisbane, QLD, Australia

**Keywords:** spoken word production, taboo words, attention capture, picture-word interference, functional magnetic resonance imaging

## Abstract

Every language has words deemed to be socially inappropriate or ‘taboo’ to utter. Taboo word production appears prominently in language disorders following brain injury. Yet, we know little about the cognitive and neural mechanisms involved in processing taboo compared to neutral language. In the present study, we introduced taboo distractor words in the picture word interference paradigm during functional magnetic resonance imaging to investigate how these words influence spoken word production. Taboo distractor words significantly slowed picture-naming latencies compared to neutral words. This interference effect was associated with increased blood oxygen level dependent signal across a distributed thalamo-cortical network including bilateral anterior cingulate cortex and left inferior frontal gyrus, left posterior middle temporal gyrus and right thalamus. We interpret our findings as being consistent with an account integrating both domain-general attention-capture/distractor blocking and language-specific mechanisms in processing taboo words during spoken word production.

## Introduction

Every language has words deemed to be taboo or ‘socially inappropriate’ to utter (e.g. profanities like swearing and cursing). The types of taboo words prohibited in different societies appear to converge on common themes, for example, sex, excrement and their related body parts (Flynn, 1976; Allan and Burridge, 2006). The universality of these taboo concepts suggests they form an integral component of the human language faculty. Uttering a taboo word in a socially inappropriate context can have a negative emotional impact, such as embarrassing the speaker and/or offending the listener. However, taboo language use is not always aligned with negative experience and can be used to impact on emotionality in a positive manner (e.g. jocular ribbing with friends, enticing a consenting partner during sex, etc.). The social utility of taboo language is highly pragmatic (Jay and Janschewitz, 2008) and context is important when attempting to ground the meaning of the intended use (MacKay *et al*., 2004; MacKay *et al*., 2015).

Taboo language features prominently in certain brain disorders. The ability to swear is frequently intact and fluent among aphasic patients (van Lancker and Cummings, 1999). Broca’s (1861) famous patient ‘Leborgne’, often described as only being able to produce the word ‘tan’, in fact augmented his limited speech output with an occasional curse, the oath ‘Sacre nom de Dieu’ (Code, 2013). The selective preservation of swearing in aphasic patients suggests taboo words comprise a unique class of words neurologically distinct from normal language use. Taboo language also features prominently in Gilles de la Tourette syndrome, a neuropsychiatric disorder characterised by chronic, involuntary tics. Coprolalia, the unprovoked production of taboo words or socially inappropriate remarks, is present in a subset of patients with Tourette’s syndrome and is often attributed to basal ganglia dysfunction disrupting inhibitory processes (Shapiro *et al*., 1998).

Jay (1999) proposed a comprehensive model of swearing that predicts the probability a taboo word will be uttered given the individual’s neurological state, psychological state and social context. The model emphasises autonomic arousal in the brain and broadly contrasts the left lateralisation of language function with right hemisphere involvement in processing emotional and taboo language. Frontal lobe damage is proposed to increase swearing while amygdala damage decreases it (Jay, 2009). The model also nominates the left frontal lobe as a likely candidate for control and inhibitory operations in taboo language production (Jay, 1999).

Using a taboo version of the SLIP task (spoonerisms of a laboratory induced predisposition; Motley *et al*., 1982), with functional magnetic resonance imaging (fMRI), Severens *et al*., (2012) reported increased blood oxygen level dependent (BOLD) signal in the right inferior frontal gyrus (IFG) that they interpreted as reflecting internal inhibition of taboo utterances. Using the same task with electroencephalography, Severens *et al*., (2011) reported an augmented negative wave arising around 600 milliseconds after the pronunciation cue for taboo trials. They proposed an account in which taboo utterances are covertly formed and monitored via an internal channel that inspects the speech plan and the right IFG inhibits them based on their social inappropriateness (e.g. Aron *et al*., 2014).

An additional source of evidence about the neurobiology of taboo language processing comes from a variant of the Stroop colour-word paradigm. When taboo or negatively valenced words are used in the Stroop task, colour-naming latencies are typically longer compared to neutral words (Siegrist, 1995; MacKay *et al*., 2004). According to binding theory (MacKay *et al*., 2004, 2015), the arousing properties of taboo words involuntarily prioritise the allocation of (i.e. captures) limited-capacity attentional resources that bind the intended meaning of the threatening stimulus to contextual features of the occurrence at the cost of primary task processing. MacKay *et al*. (2004, 2015) proposed the priority binding of taboo words is accomplished via word-specific links between the amygdala and hippocampus.

Song *et al*. (2017) recently conducted a meta-analysis of 16 neuroimaging studies that employed the emotional Stroop task. An activation likelihood estimate analysis contrasting emotional > conventional versions of the Stroop task identified increased activation spanning the left lateral prefrontal cortex (PFC), the left medial PFC and the left dorsal anterior cingulate cortex (ACC). These areas were reliably activated in the conventional Stroop but showed a greater response magnitude for the emotional contrast. Song *et al*. (2017) proposed the left dorsolateral PFC mediates the resolution of stimulus conflict via a selective attention mechanism, while the left IFG performs top-down suppression of emotional conflict (e.g. Jay, 1999). The dorsal subdivision of the ACC is often implicated in cognitive/lexical conflict and control, whereas the ventral portion of the ACC is more commonly linked with emotional conflict and control (Mohanty *et al*., 2007; Piai *et al*., 2013). Interestingly, no reliable activation was observed in the ventral portion of the ACC or in the amygdala, suggesting these regions might not be critical for the resolution of emotional interference during speech production compared to comprehension tasks (cf. MacKay *et al*., 2015).

Taboo effects during speech production have also been demonstrated using the picture-word interference paradigm (PWI: Rosinski *et al*., 1975) but are yet to be investigated with neuroimaging. The PWI paradigm requires participants to name pictures of neutral objects while ignoring accompanying distractor words. Taboo distractor words reliably slow picture-naming latencies compared to neutral distractor words (Dhooge and Hartsuiker, 2011; White, *et al*., 2016; Hansen, *et al*., 2017). Using a speeded version of the taboo PWI task in which participants are instructed to prioritise speed over accuracy, Dhooge and Hartsuiker (2011) observed fewer naming errors for taboo compared to neutral trials. Like Severens *et al*. (2011, 2012), they proposed a post-lexical account based on the inner speech channel of Levelt’s (1989) influential perceptual loop theory in which the verbal self-monitor is considered to be sensitive to the social appropriateness of response-level representations in an output buffer. Upon detection of a taboo distractor word in the buffer, the self-monitor increases scrutiny of the subsequent target response, slowing its production.

Other researchers have proposed the taboo PWI effect might instead reflect the operation of pre-lexical or lexical-level mechanisms. Slevc and Ferreira (2006) propose taboo interference might be better explained by the highly arousing properties of taboo distractors capturing limited attentional resources at the expense of primary task processing. Hansen *et al*. (2017) and White *et al*. (2016) demonstrated taboo interference is reduced or eliminated when distractors share an initial phoneme with the target picture name (i.e. phonological facilitation). This finding is inconsistent with a post-lexical self-monitoring account, as response-level representations only occupy the output buffer *after* phonological encoding is completed (cf. Dhooge and Hartsuiker, 2011). Hansen *et al*. (2017) also showed taboo interference survives distractor masking that is assumed to prevent phonologically well-formed representations entering an output buffer. The authors concluded their results were best explained by an attention-capture account (e.g. Slevc and Ferreira, 2006; Röer *et al*., 2017). Alternatively, White *et al*. (2016) invoke the distractor-blocking mechanism in the WEAVER++ computational model of spoken word production (Roelofs, 2003, 2008) to explain taboo interference in PWI. According to this account, the arousing nature of taboo words enhances their lexical activation requiring a distractor-blocking mechanism to intervene and suppress their further processing. Taboo words are blocked more slowly due to their capturing limited attentional resources also required by the blocking mechanism. Attentional control of speech production in the WEAVER++ model is regulated by condition-action rules embedded within an executive control network proposed to involve the left ACC and lateral PFC (Roelofs, 2003, 2008).

The present study tested rival accounts of taboo interference effects in spoken word production using the PWI paradigm and converging evidence from fMRI. A sparse imaging design was adopted to allow the collection of overt verbal responses in the absence of scanner noise (Eden *et al*., 1999; Elliott *et al*., 1999). Thus far, evidence from taboo SLIP and emotional Stroop tasks reliably implicates an anterior network of brain regions including left and right IFG, left dorsolateral PFC and the dorsal ACC in taboo word interference (Severens *et al*., 2012; Song *et al*., 2017; see also Jay, 1999). However, the evidence concerning roles for the amygdala and the ‘emotional’ ventral subdivision of the ACC is less compelling (cf. Jay, 1999; MacKay *et al*., 2015). According to the post-lexical account (Dhooge and Hartsuiker, 2011), the taboo effect in PWI is due to the verbal self-monitor and an inhibitory control mechanism operating on response-level representations in an output buffer, i.e. the account implicates both picture naming/production and domain-general control processes. Indefrey’s (2011; see also Indefrey and Levelt, 2004) updated meta-analysis of neuroimaging studies of speech production ascribed roles for the left superior temporal gyrus (STG) in verbal self-monitoring and the left IFG and supplementary motor area (SMA) in response-level processing such as phonetic encoding and syllabification (*vs* motor articulation). Hence, we predict involvement of these regions and the right IFG (Severens *et al*., 2012) if the post-lexical account is correct.

Alternatively, the taboo effect might have a lexical locus (Slevc and Ferreira, 2006; White *et al*., 2016; Hansen *et al*., 2017). This account also implicates picture naming/production processes in the taboo interference effect. Indefrey’s (2011) meta-analysis ascribed lexical-level processing to the mid portion of the left middle temporal gyrus (MTG, grammatical encoding and lemma selection) and the posterior portions of the MTG and STG (phonological word-form encoding and lexeme retrieval). Yet another possibility is that an attentional distractor-blocking mechanism is involved due to the arousal properties of taboo words capturing attention at the expense of the primary task. However, this distractor-based attention-capture/blocking mechanism could be relatively independent of the production system, i.e. it could be engaged irrespective of the nature of the primary task. For example, a recent study by Mädebach *et al*., (2018) found that taboo distractors not only interfere with picture naming but also impede manual responses for phoneme decisions and natural size judgements about depicted objects. If so, then dorsolateral PFC, IFG and ACC (dorsal subdivision) might be engaged (see Song *et al*., 2017). Although the right IFG is often proposed to play a role in inhibitory control (e.g. Severens *et al*., 2012), other researchers have also ascribed it a role in attention capture by distracting information (Sharp *et al*., 2010; de Fockert and Theeuwes, 2012; see Aron *et al*., 2014 for discussion). However, it has also been proposed that taboo words’ arousal properties might enhance lexical activation such that domain-general attention-based operations of a distractor-blocking mechanism are then applied to intervene and suppress further processing (see Roelofs, 2003, 2008; White *et al*., 2016). If so, we would expect to observe differential activity in regions responsible for both lexical-level processing and attention-capture/blocking mechanisms.

We also included a manipulation of phonological relatedness between taboo distractors and target pictures, as this has been shown to attenuate the taboo interference effect in PWI (White *et al*., 2016; Hansen *et al*., 2017). White *et al*. (2016) hypothesised if heightened attention facilitates phonological encoding, then greater phonological facilitation would be expected for taboo words that are particularly attention-grabbing, i.e. taboo interference and phonological facilitation interact. However, using additive factors logic, Hansen *et al*. (2017) showed phonological facilitation and taboo interference effects are additive, indicating the effects are localized at different processing stages in word production, i.e. taboo interference occurs either earlier or later than word-form retrieval. We therefore included the phonological manipulation to test which of these explanations is correct. If phonological facilitation and taboo interference interact due to attention capture, then we expect differential activity in the posterior portions of the MTG and STG (phonological word-form encoding/lexeme retrieval) and the dorsolateral PFC, IFG and ACC (attention capture/blocking). If taboo interference instead arises at an earlier or later stage than phonological facilitation, then we expect the phonological manipulation will result in differential activity only in the posterior portions of the MTG and STG (phonological word-form encoding/lexeme retrieval).

## Method

### Participants

We recruited 23 healthy volunteers (11 female, 12 male) aged from 18 to 34 years (M = 23.35, s.d. = 4.50). Each participant provided written informed consent and was compensated AUD$30 for their time and effort. All participants identified as monolingual English speakers, right-handed, with normal or corrected-to-normal vision and no history of neurological or psychiatric disorder. All participants were warned about the potentially offensive/embarrassing nature of the taboo stimuli to be used in the experiment both at the time of online sign-up and again upon arrival at the experiment and informed they were free to withdraw at any time without penalty. The study was approved by the Ethical Committee of the University of Queensland. We based our sample size on prior fMRI studies of interference and phonological effects with comparable effect sizes in PWI paradigms in our and others’ laboratories (de Zubicaray *et al*. 2002; de Zubicaray *et al*., 2013; Piai *et al*., 2013; Muehlhaus *et al*., 2014).

### Behavioural design

This experiment used a repeated measures design. The independent variable was distractor word type (within-participant and within-item) with the following three levels: neutral, taboo and phonologically related taboo. Dependent variables were target picture-naming latency (in milliseconds) and naming accuracy.

### Stimuli and materials

Twenty-five black and white line drawings depicting familiar objects were used as target picture stimuli, the majority of which were selected from normative picture databases (Cycowicz *et al*., 1997; Szekely *et al*., 2004) with remaining items selected from the internet. Fifty nouns were used as distractors paired with unrelated target pictures. We selected the 24 taboo words with the highest taboo ratings (i.e. profanities, vulgarities, slurs, etc.) and matched 25 neutral words according to normative data provided by Janschewitz (2008). Data for an additional taboo word (*wanker*) not included in Janschewitz (2008) were taken from Eilola and Havelka’s (2010) normative study that used comparable rating scales. Each picture was paired with two unrelated neutral words and two taboo words. One picture and taboo word pairing was phonologically unrelated. The other picture and taboo word pairing minimally shared an initial phoneme (see Supplementary Materials for stimuli lists). Distractor words were not part of the target picture response set, nor were they semantically related to their paired pictures.

Taboo and neutral words were matched closely on a range of lexical properties including frequency, number of letters, phonemes, syllables and neighbours, in addition to word mean bigram frequency, familiarity and imageability. Taboo words scored lower on ratings of valence (rated from negative to positive) and higher on ratings of taboo status, offensiveness and arousal (see Supplementary Materials for table of lexical properties).

### Apparatus and procedure

Stimulus presentation, response recording and latency measurement (i.e. voice key) were accomplished via the Cogent 2000 toolbox extension (www.vislab.ucl.ac.uk/cogent_2000.php) for MATLAB (2010a, MathWorks Inc.). Naming responses were recorded on digital audio files using a custom positioned fibre-optic dual-channel noise-cancelling microphone (FOMR-III, Optoacoustics Ltd., Or-Yehuda, Israel; www.optoacoustics.com) attached to the head coil. Naming latencies were calculated automatically with custom-written voice key software in Cogent and verified using Audacity software in case non-vocal noise triggered the key.

A PWI paradigm was employed. After placement in the scanner, participants first underwent picture familiarisation followed by the experiment proper. Picture familiarisation involved participants naming each of the 25 pictures (presented in a random order) in three separate runs. The first run presented pictures with their correct name printed beneath for reference if needed. Subsequent runs presented only the pictures. The experimenter corrected any naming errors.

For the experiment proper, participants were instructed to name the pictures aloud as quickly as possible and ignore any distractor words. They were asked not to speak or move during image acquisition and in the event of a naming error not to correct their response. Target pictures were presented centrally on a white background. Distractor words were presented centrally in lower case red Arial font. Stimuli were back projected onto a screen that the participant viewed through a mirror mounted on the head coil. The size of the pictures was ~10 × 10 centimetres and subtended ~10^°^ of visual angle when each participant was in position for imaging. Participants completed two blocks of 50 picture-naming trials for a total of 100 trials. During a short break between blocks, a structural scan was acquired (see Image acquisition). Participants viewed each of the 25 pictures four times, paired with two neutral distractors, one phonologically unrelated taboo distractor and one phonologically related taboo distractor. The order of the trials was pseudorandomised across participants using Mix software (van Casteren and Davis, 2006) such that two presentations of the same picture were always interceded by at least five different pictures, and no more than two successive trials were from the same distractor type condition.

Each trial began with a black fixation point (+) presented centrally for 500 milliseconds followed by a blank screen for 500 milliseconds. Next the target picture and distractor word were presented for 750 milliseconds, followed by a blank screen for 13.75 s. An image volume was acquired 4 s following the presentation of the target picture (see Image acquisition). The next trial commenced after an inter-trial interval of 15 s.

#### Image acquisition

Images were acquired using a 3T MAGNETOM Trio TIM MRI system (Siemens Medical Solutions, Erlangen, Germany) with a 12-channel Matrix head coil. Functional T2^*^-weighted images depicting BOLD contrast were acquired using a gradient echo echo-planar imaging (EPI) sequence (36 slices, TA 3000 milliseconds, TR 15000 milliseconds, TE 36 milliseconds, 64 × 64 matrix, 3.3 × 3.3 millimetre in plane resolution, 3 millimetre slice thickness with 0.3 millimetre gap and flip angle 80^°^). A point spread function mapping sequence was acquired prior to the EPI data to correct geometric distortions (Zaitsev *et al*., 2004). Behavioural trials were interleaved with detection of BOLD signal changes using a sparse acquisition method in which a single image volume was acquired coincident with the estimated peak hemodynamic response, allowing overt naming responses to be recorded in the absence of gradient scanner noise. Two blocks of 50 trials were acquired. A 30-decibel attenuating headset was used to reduce gradient noise. A 3D T1-weighted structural image was also acquired using a magnetization prepared rapid acquisition gradient echo sequence (1 millimetre isotropic voxels). Total imaging time was ~30 min.

#### Image analysis

Image processing and statistical analyses were performed using statistical parametric mapping software (SPM12, Wellcome Trust Centre for Neuroimaging). The first volume in each fMRI block was discarded. Remaining images were motion corrected using the INRIalign toolbox (Freire *et al*., 2002). A mean image was generated from the realigned series and co-registered to the T1-weighted image. The T1-weighted image was next segmented using the ‘Segment’ routine. The DARTEL toolbox (Ashburner, 2007) was then employed to create a custom group template from the segmented grey and white matter images, and individual flow fields were used to normalize the realigned fMRI volumes to the Montreal Neurological Institute (MNI) atlas T1 template. The images were resampled to 2 millimetre^3^ voxels and smoothed with a 8 millimetre full-width half-maximum isotropic Gaussian kernel. Global signal effects were then estimated and removed using a voxel-level linear model (Macey *et al*., 2004). In a final step, the images from each distractor condition were regrouped to form a single epoch and errors/omissions assigned to a separate condition. Low and high pass filtering were not applied due to the long TR (15 seconds).

We conducted a two-stage, mixed-effects model statistical analysis. Epoch types corresponding to each distractor and error condition were modelled as effects of interest with delta functions representing each epoch onset and convolved with a basis function consisting of a single finite impulse response corresponding to a boxcar function that encompassed the epoch length. As the sparse image sequence does not acquire BOLD time course information, trials were not convolved with a conventional hemodynamic response function (see Eden *et al*., 1999; Elliott *et al*., 1999; Gracco *et al*., 2005). Linear contrasts were applied to each participant’s parameter estimates at the fixed-effects level and then entered in a group-level random effects repeated measures analysis of variance in which covariance components were estimated using a restricted maximum likelihood procedure to correct for nonsphericity (Friston *et al*., 2002). Planned *t* contrasts were employed to identify regions showing significant differences in activity among the three experimental conditions.

As we had a priori hypotheses concerning specific neuroanatomical regions associated with various mechanisms involved in speech production and taboo word processing, we opted to first restrict voxel-wise analyses to a set of predefined regions of interest (ROIs) via small volume corrections (SVCs), thereby controlling for multiple comparisons only in those voxels, using labelled maximum likelihood maps from three-dimensional probabilistic atlases. We used Hammers’ *et al*. (2003) probabilistic atlas as it encompassed the stereotactic MNI co-ordinates reported. We pre-defined the following ROIs: left IFG (inhibitory control of emotional conflict: Jay, 1999; Song *et al*., 2017; response-level syllabification and phonetic encoding: Indefrey, 2011), right IFG (inhibitory control: Severens *et al*., 2012; Aron *et al*. 2014; attention capture: Sharp *et al*., 2010; de Fockert and Theeuwes, 2012), ACC (executive control of speech production: Roelofs, 2003, 2008) with further subdivision into dorsal ACC reflecting lexical/cognitive conflict and ventral ACC reflecting emotional conflict (Mohanty *et al*., 2007; Piai *et al*., 2013; Song *et al*., 2017), left STG (self-monitoring: Indefrey, 2011), left mid portion of the MTG (grammatical encoding and lemma selection: Indefrey, 2011), left posterior MTG and STG (phonological word-form encoding and lexeme retrieval: de Zubicaray *et al*., 2002; Indefrey, 2011) and amygdala (arousal/emotional valence: MacKay *et al*., 2015). The ROI analyses were followed by an exploratory whole-brain analysis. For both analyses, we applied a height threshold of *P* < 0.001 and family-wise error corrected cluster threshold of *P* < 0.05.

## Results

### Behavioural data

Data from one participant was excluded due to a technical difficulty resulting in incomplete image acquisition. Data from an additional participant was excluded for generating excessive (27%) speech dysfluencies immediately prior to naming (i.e. tisking or tutting). An additional two participants were excluded due to excessive head movement during image acquisition, defined as motion exceeding one voxel (3 millimetres) within a single imaging block.

For the remaining 19 participants, non-speech noises and technical errors (voice key malfunctions) accounted for 0.37% of the total data and were excluded from further analysis. Participant naming errors (for example, incorrect responses, verbal dysfluencies) accounted for 2.11% of the total data were likewise excluded and due to their low frequency, were not further analysed. Naming latencies more than three standard deviations from each participant’s individual mean, calculated within condition, were also removed and accounted for 1.74% of the total data. Mean-naming latencies as a function of distractor type are reported in Table 1.

**Table 1:** Mean naming latencies in milliseconds (with 95% confidence intervals in parentheses) and error rates as a function of distractor type

	*Distractor type*		
	*Neutral*	*Taboo*	*Phonologically related taboo*
	*M*	*M*	*M*
*Naming latency*	878 (±16)	972 (±22)	985 (±22)
*Error rate*	*2.11%*	*2.11%*	*2.11%*
*Taboo effect*		94 (±38)	107 (±43)
*Phonology effect*			13 (±20)

*Note*: Taboo effect calculated as mean taboo/phonologically related taboo minus mean neutral naming latency. Phonology effect calculated as mean phonologically related taboo minus mean taboo naming.

A repeated measures MANOVA (O’Brien and Kaiser, 1985) with participants (*F*_1_) and items (*F*_2_) as random factors revealed a significant main effect of distractor type by both participants and items [*F*_1_ (2, 17) = 13.67, *P* < 0.001, $\eta^{\,2}_{\mathrm{p}}$ = 0.62 and *F*_2_ (2, 23) = 57.55, *P* < .001, $\eta^{\, 2}_{\mathrm{p}}$ = 0.83]. Planned comparisons (one-tailed paired *t*-tests) revealed that the average naming latency for the taboo condition was significantly slower than the neutral condition by ~94 milliseconds [*t*_1_ (18) = −5.20, *P* < 0.001, *d* = 0.56 and *t*_2_ (24) = −6.54, *P* < 0.001, *d* = 1.25]. The average naming latency for the phonologically related taboo condition was also significantly slower than the neutral condition by ~107 milliseconds [*t*_1_ (18) = −5.25, *P* < 0.001, *d* = 0.64 and *t*_2_ (24) = −8.65, *P* < 0.001, *d* = 1.64]. Finally, the average naming latencies for the taboo and phonologically related taboo conditions did not differ significantly [*t*_1_ (18) = −1.50, *P* = 0.152, *d* = 0.07 and *t*_2_ (24) = −0.55, *P* = 0.585, *d* = 0.12].

### Imaging data

#### A priori defined ROI analyses

The *t* contrast of phonologically related taboo > taboo distractor conditions revealed no significant activity in *any* of the a priori defined ROIs, mirroring our behavioural results. Additionally, no significant activity was observed in any of the ROIs for the reverse contrast (taboo > phonologically related taboo). Given the absence of either behavioural or fMRI effects for this contrast, we collapsed both phonologically related taboo and taboo conditions to create an overall taboo condition and contrasted this with the neutral distractor condition in subsequent analyses.

The *t* contrast of all taboo > neutral distractor conditions revealed significant activity in the left IFG, bilateral ACC and left posterior temporal lobe ROIs, the latter with a peak in the MTG. No significant activity was observed in the mid portion of the left MTG, left STG or amygdala. The reverse contrast (neutral > all taboo) revealed no significant activity in any of the a priori defined ROIs using SVCs (Table 2).

**Table 2:** Cerebral regions showing significant activity for distractor type comparisons

Contrast	Peak			Z-score	Cluster size (voxels)
	x	y	z		
*All taboo > neutral*					
Left IFG (P Tri)^b^	−44	18	8	4.75	529
	−42	18	26	4.13	90
Left ACC^a^^,^^b^	0	16	28	5.94	7815
Right ACC^b^	2	14	30	5.81	871
Left posterior MTG^a^^,^^b^	−52	−58	12	5.41	2373
Right MTG^a^	56	−54	14	5.39	1140
Left insula^a^	−38	16	−14	5.44	1461
Right insula^a^	32	10	−10	4.23	277
Right MFG^a^	38	4	38	4.7	435
Right postcentral gyrus^a^	36	−38	68	4.26	217
Right thalamus^a^	6	6	12	4.96	743
*Neutral > all taboo*					
Right calcarine gyrus^a^	24	−84	8	4.81	307

*Note.* Height threshold *P* < 0.001 and *P* < 0.05 cluster FWE corrected.

^a^Whole-brain corrected.

^b^Small volume corrected.

#### Unrestricted whole-brain analyses

**Fig. 1:**
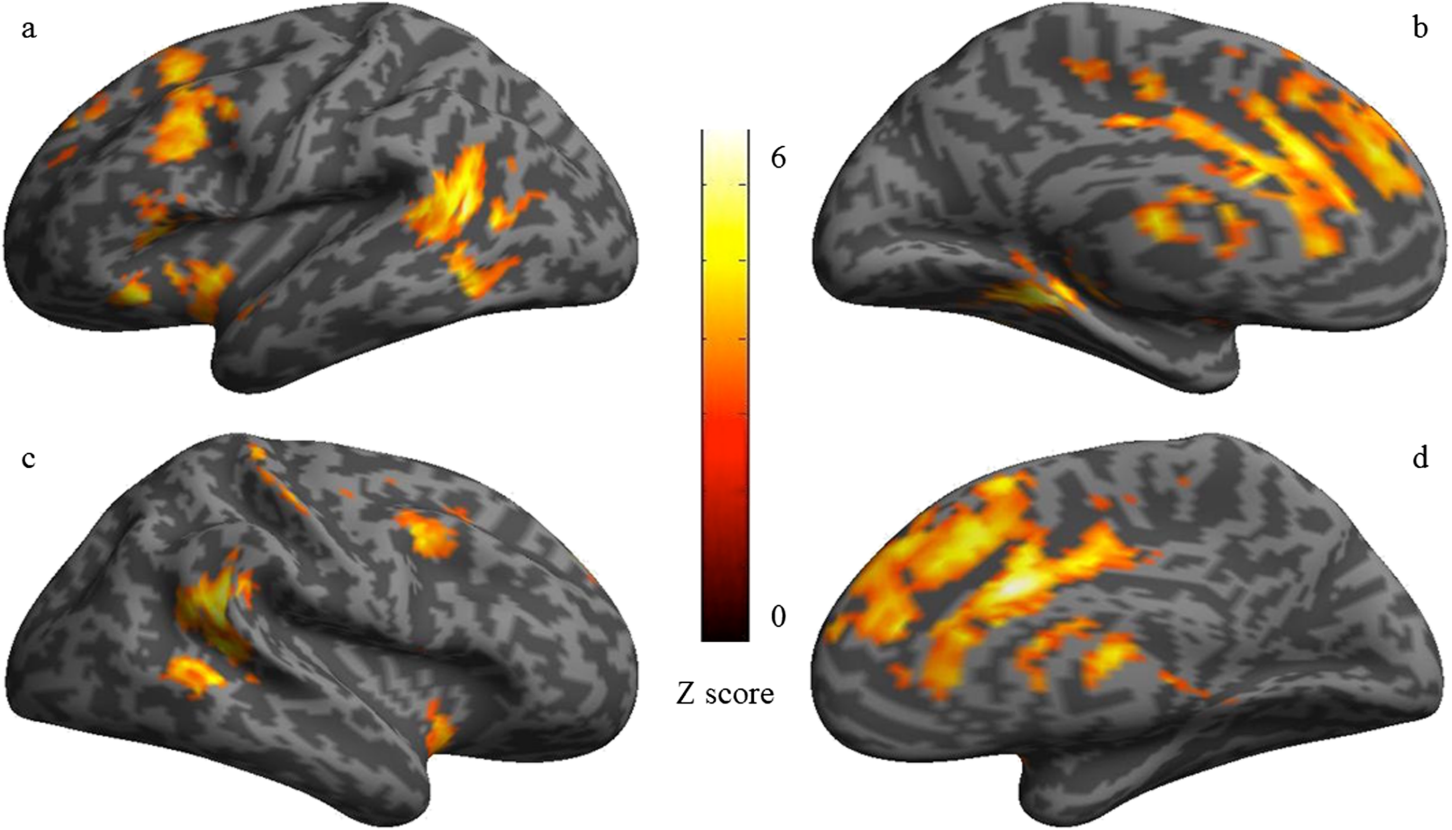
Cerebral regions showing significant activity for the taboo interference effect (all taboo > neutral) in the whole-brain analysis superimposed on the inflated surface rendering of an individual brain [height threshold *P* < 0.001and cluster threshold *P* < 0.05 family-wise error (FWE) corrected = 217 voxels]. (**A**) Left hemisphere lateral view, (**B**) left hemisphere medial view, (**C**) right hemisphere lateral view and (**D**) right hemisphere medial view.

The *t* contrast of all taboo > neutral distractor conditions revealed significant activity in eight large clusters (Figure 1). As Table 2 shows, the same regions revealed in the ROI analyses were also significant in the whole-brain analysis. Activation in a large bilateral cluster with a peak in the left ACC extended into the premotor cortex/precentral gyrus. Activation was also observed in the left posterior MTG and in the left insula-operculum including IFG. Activation was also observed in the right hemisphere homologues of the posterior MTG and STG and the right middle frontal gyrus (MFG). Additional activity was observed in the right post central gyrus and the thalamus.

## Discussion

This study investigated the cognitive and neural mechanisms responsible for taboo language processing in spoken word production by testing alternative accounts of how taboo distractor words influence PWI effects. We replicated the taboo interference effect in PWI and, for the first time, identified a large-scale, thalamo-cortical network underlying it. As predicted, naming latencies were significantly slower for the taboo compared with the neutral condition. The magnitude of this interference effect was comparable to previous reports (Dhooge and Hartsuiker, 2011; White *et al*., 2016; Hansen *et al*., 2017). Surprisingly, naming latencies did not significantly differ between taboo and phonologically related taboo conditions. Previous studies reported a facilitation effect for phonologically related distractors such that taboo interference was reduced or eliminated (de Zubicaray *et al*., 2002; White *et al*., 2016; Hansen *et al*. 2017). The fMRI data likewise revealed no differential activity in any of the ROIs for the taboo > phonologically related taboo contrast and vice-versa. We discuss a potential explanation for this null result below.

Given the null result for the phonological manipulation, we pooled the taboo and phonologically related taboo conditions to create an ‘all taboo’ condition which we then contrasted with the neutral condition. This ‘all taboo > neutral’ contrast identified significant differential BOLD activity across a distributed cortical network including all of our a priori defined ROIs except for the mid portion of the left MTG, left STG, right IFG and amygdala. The increased BOLD signal identified in the medial frontal cortex formed a large bilateral cluster with its peak in the left ACC but also spanning the right ACC. The increased BOLD signal in the left PFC was part of a cluster that also included the insula and left IFG. Differential activity was also observed in the right hemisphere homologues including the right insula and MFG and in the thalamus.

To what extent is the observed activity compatible with contemporary neurobiological accounts of taboo language processing? Our findings appear only partly compatible with the post-lexical account of taboo interference in PWI in which verbal self-monitoring and inhibitory control are proposed to be engaged (Dhooge and Hartsuiker, 2011; Severens *et al*., 2011, 2012). We observed activity in the left posterior temporal lobe that has been ascribed a role in verbal self-monitoring across various studies (for review, see Indefrey, 2011). However, the activity was part of a larger cluster with its peak in the posterior MTG that has also been ascribed a role in lexical-level processing (lexeme retrieval; Indefrey, 2011). Our data also revealed differential activity in the left IFG and SMA, supporting the involvement of post-lexical phonetic encoding and syllabification mechanisms (Indefrey, 2011). However, Severens *et al*.’s (2012) fMRI study of the taboo SLIP task suggested a role for the right IFG in inhibiting taboo words, yet we did not observe significant activity in this region for taboo *vs* neutral distractors.

Our data appear mostly compatible with an account that combines attentional and lexical-level mechanisms (Slevc and Ferreira, 2006; White *et al*., 2016; Hansen *et al*., 2017; Röer *et al*., 2017). Interestingly, we observed significant differential activation in the thalamus in the whole-brain analysis. A role for the thalamus in arousal is well documented, but a specific link with the arousal properties of individual words is less well established (see Llano, 2013, for review). Together with the significant activity in the left posterior MTG, ACC and IFG, thalamic involvement is consistent with an account in which taboo words’ arousal properties enhance lexical activation such that domain-general attention-based operations of a distractor-blocking mechanism are then applied to intervene and suppress further processing (Roelofs, 2003, 2008; White *et al*., 2016). Taboo words, while detected early, are blocked more slowly due to their capturing limited attentional resources also required by the blocking mechanism. Hansen *et al*. (2017), in an earlier behavioural study of taboo interference in PWI, found support for taboo interference arising early in speech production.

By contrast, several accounts have emphasised key roles for brain regions frequently implicated in emotional processing, such as the amygdala or ventral ACC, in taboo language (Jay, 1999; MacKay *et al*., 2015). Neither of these regions showed significant differential activity in the present study despite the use of sensitive ROI analyses. One possibility is that taboo words comprise a distinct linguistic class compared to negative emotion words, as some linguists have suggested (e.g. Jay, 1999). For example, the social utility of taboo language is highly pragmatic. A number of researchers have noted taboo language use is not always aligned with negative experience unlike other negatively valenced words and can be used to impact on emotionality in a positive manner (see Jay and Janschewitz, 2008). Alternatively, as Song *et al*. (2017) proposed, emotional valence may play a less prominent role in spoken word production compared to comprehension tasks.

While we favour an arousal/attention-capture explanation of the current results, one caveat concerns our failure to replicate the phonological facilitation effect reported in previous studies (White *et al*., 2016; Hansen *et al*., 2017). While we employed the same stimuli as our earlier study, there was one departure in methodology that might explain the absence of this effect in our current study. Our previous experiment ensured the presentation of the distractor word was not in a predictable location/order. Due to the constraints of the mirror mounted on the MRI system’s head coil, we opted to present distractor words in a fixed central position. However, Roelofs and Piai (2011) have shown distractors with fixed spatial position can be blocked more quickly than distractors with variable spatial positioning.

How might faster blocking of centrally presented distractor words eliminate the phonological facilitation effect while preserving taboo interference? There is some evidence to indicate that taboo processing occurs earlier and is more automatic than word-form retrieval. In our earlier study (Hansen *et al*., 2017), we showed taboo interference survives distractor masking while phonological facilitation does not. Another recent PWI study demonstrated precues (i.e. alerting participants to the nature of an upcoming trial) were ineffective in reducing taboo interference when taboo trials were mixed with non-precue trials (White *et al*., 2018). Thus, taboo word processing appears to involve a relatively automatic (or involuntary) arousal-related attention-capture mechanism. This explanation, while *post hoc*, is at least consistent with the attention-capture account as it does not require additional assumptions.

This is the first fMRI study to investigate the taboo interference effect in spoken word production. We successfully replicated the taboo interference effect in PWI and provided evidence for the involvement of a distributed thalamo-cortical network. The cortical areas we identified are potentially consistent with both post-lexical monitoring/inhibition and domain-general attention-capture accounts of taboo interference, although we favour the latter explanation given the differential activation of the thalamus and converging behavioural evidence for an early rather than late post-lexical effect and failure to observe right IFG activity. However, we were unable to find evidence supporting the involvement of brain regions associated with emotional processing, such as the amygdala and ventral ACC. This might indicate taboo words constitute a distinct class of words compared to negative emotion words or that emotional valence is relatively less engaged in production than comprehension.

## Supplementary Material

Supplementary Data
