# Supplementary material for: The neurobiology of taboo language processing: fMRI evidence during spoken word production
Source: Soc Cogn Affect Neurosci. 2019 Feb 1;14(3):271–9. doi: 10.1093/scan/nsz009 (PMC6399611; doi:10.1093/scan/nsz009)
Supplement: Supplementary Data [file nsz009_supp.zip › scan-18-214-File007.docx]

Supplementary Table 1

Target picture and distractor word pairings for neutral, taboo and phonologically related taboo conditions.

|  | *Distractor Word Type* | | | |
| --- | --- | --- | --- | --- |
| *Picture* | *Neutral (1)* | *Neutral (2)* | *Taboo* | *Phonologically Related Taboo* |
| ASTRONAUT | chaos | clock | slut | asshole |
| BATH | gender | stairs | cocksucker | bastard |
| BIN | fame | song | twat | bitch |
| BLOUSE | ship | range | wanker | blowjob |
| BOOTS | chair | wit | motherfucker | boobs |
| BOW | den | rake | dildo | boner |
| CLIPBOARD | rake | den | asshole | clit |
| COLLAR | adventure | debt | prick | cocksucker |
| COMPUTER | passage | fame | pussy | cum |
| CRAB | fraud | theory | nigger | crap |
| CUP | tower | phase | shithead | cunt |
| DINGHY | penalty | chaos | retard | dildo |
| DISHWASHER | song | peace | bitch | dick |
| FAN | wit | tower | tits | fag |
| HORSE | stairs | passage | bastard | whore |
| MOTH | radiator | chair | boobs | motherfucker |
| NEEDLE | theory | fraud | crap | nigger |
| PRINTER | debt | adventure | whore | prick |
| PURSE | writer | gender | dick | pussy |
| RECORDER | headache | penalty | clit | retard |
| SHELL | peace | writer | cum | shithead |
| SLUG | clock | radiator | boner | slut |
| TISSUES | range | ship | blowjob | tits |
| TWEEZERS | phase | item | cunt | twat |
| WAVES | item | headache | fag | wanker |

As used in Hansen et al. (2017).
