# Supplementary material for: The neurobiology of taboo language processing: fMRI evidence during spoken word production
Source: Soc Cogn Affect Neurosci. 2019 Feb 1;14(3):271–9. doi: 10.1093/scan/nsz009 (PMC6399611; doi:10.1093/scan/nsz009)
Supplement: Supplementary Data [file nsz009_supp.zip › scan-18-214-File008.docx]

Supplementary Table 2

Lexical properties of the neutral and taboo distractor words.

|  | *Distractor Word Type* | | | |
| --- | --- | --- | --- | --- |
|  | *Neutral* | | *Taboo* | |
| *Lexical Property* | *M* | *SD* | *M* | *SD* |
| Letters | 5.32 | (1.55) | 5.52 | (2.10) |
| Phonemes | 4.08 | (1.26) | 4.56 | (1.66) |
| Syllables | 1.60 | (0.82) | 1.60 | (0.76) |
| Neighbours | 18.76 | (16.86) | 19.56 | (19.54) |
| Bigram Mean | 3545.33 | (1580.74) | 3363.29 | (1387.94) |
| Frequency | 24.90 | (27.47) | 24.54 | (37.11) |
| Familiarity | 5.31 | (0.91) | 5.13 | (1.11) |
| Imageability | 4.59 | (2.15) | 4.57 | (1.68) |
| Taboo Status | 1.15 | (0.26) | 6.15 | (1.08) |
| Offensiveness | 1.09 | (0.21) | 3.79 | (1.23) |
| Valence | 4.96 | (1.06) | 2.91 | (1.06) |
| Arousal | 2.04 | (0.81) | 5.18 | (0.79) |

*Note*: Ratings for familiarity, imageability, taboo status, offensiveness, valence, and arousal taken from Janschewitz (2008) and Eilola and Havelka (2010). Higher valence ratings correspond to more positive items (5 = neutral valence). All other data taken from eLexicon (Balota et al., 2007). As used in Hansen et al. (2017).
